# Characteristics of vocal cues, facial action units, and emotions that distinguish high from low self-protection participants engaged in self-protective response to self-criticizing

**DOI:** 10.3389/fpsyg.2024.1363993

**Published:** 2025-01-15

**Authors:** Viktória Vráblová, Júlia Halamová

**Affiliations:** Faculty of Social and Economic Sciences, Institute of Applied Psychology, Comenius University in Bratislava, Bratislava, Slovakia

**Keywords:** action units, emotions, self-protection, two chair technique, voice units, vocal cues

## Abstract

**Introduction:**

Self-protection, also called protective anger or assertive anger, is a key factor in mental health. Thus, far, researchers have focused mainly on the qualitative analysis of self-protection.

**Methods:**

Therefore, we investigated facial action units, emotions, and vocal cues in low and high self-protective groups of participants in order to detect any differences. The total sample consisted of 239 participants. Using the Performance factor in the Short version of the Scale for Interpersonal Behavior (lower 15th percentile and upper 15th percentile) we selected 33 high self-protective participants (11 men, 22 women) and 25 low self-protective participants (eight men, 17 women). The self-protective dialogue was recorded using the two-chair technique script from Emotion Focused Therapy. The subsequent analysis was performed using iMotions software (for action units and emotions) and Praat software (for vocal cues of pitch and intensity). We used multilevel models in program R for the statistical analysis.

**Results:**

Compared to low self-protective participants, high self-protective participants exhibited more contempt and fear and less surprise and joy. Compared to low self-protective participants, high self-protective participants expressed the action units the following action units less often: Mouth Open (AU25), Smile (AU12), Brow Raise (AU2), Cheek Raise (AU6), Inner Brow Raise (AU1), and more often Brow Furrow (AU4), Chin Raise (AU17), Smirk (AU12), Upper Lip Raise (AU10), and Nose Wrinkle (AU9). We found no differences between the two groups in the use of vocal cues.

**Discussion:**

These findings bring us closer to understanding and diagnosing self-protection.

## Introduction

### Self-protection and a link to self-criticism

In Emotion-Focused Therapy (EFT; Greenberg, 2002) self-protection is also known as assertive anger or protective anger (Pascual-Leone and Greenberg, 2007). Several scholars distinguish anger based on its productivity (Kramer et al., 2016; Pascual-Leone and Greenberg, 2007). Pascual-Leone and Paivio (2013) discuss primary (adaptive) and secondary (maladaptive) anger. Protective anger, referred to by Pascual-Leone and Greenberg (2007), is adaptive anger which is a reaction to mistreatment (e.g., when an individual's safety is violated) and to unsatisfied needs (Halamova et al., 2019). Lower self-esteem can reduce the likelihood of constructive (assertive/protective) anger forming (Pascual-Leone and Greenberg, 2007). Through self-protection, people can establish their own boundaries, stand up for themselves, and assert their rights. He et al. (2012) think anger is useful because it supports efforts to overcome obstacles, encourages motivation, and thus promotes persistence. Assertive anger is caused by limiting situations that prevent the person from fulfilling their goals. Kramer et al. (2016) confirm that assertive anger is both helpful and functional. It supports prosperity and good mental and physical health. In addition, this anger is empowering, healing, and helps improve emotional resilience and flexibility (Pascual-Leone, 2009; Halamova, 2013). That means it helps us fight emotional distress and promotes good mental health (Pascual-Leone and Greenberg, 2007). In our understanding, the construct is called differently, but we believe that various authors define the same theory. Therefore, in this study we call this constructive type of anger as “self-protection.”

Self-protection (as states its name) protects against negative self-view conducted by inner voice of an individual, typically occurring in situations of failure (Shahar, 2015). This obstructive treatment that the self-protection fights against, is also called self-criticism (Whelton et al., 2007). Since intense self-criticism is widely believed to be an unhealthy pathological trait that needs to be reduced (Falconer et al., 2015), we wanted to focus on improving the efficiency on how the therapeutic approach (with the use of the self-protection) can be used to lower the levels of self-criticism.

### Emotions, facial expressions, action units, and coding

Emotions and facial expressions were broadly studied by Ekman (2003), which led to the creation of the Facial Action Coding System (FACS; Ekman and Friesen, 1978; Ekman, 2002). The FACS describes the movements of facial muscles (anatomical facial movements), which are composed of “action units” (AU; Ekman and Friesen, 1978). Ekman (2003) also identified seven universal emotions (universal across cultures)—fear, disgust, joy, sadness, anger, contempt, and surprise, which are all distinctive but share related action units. These studies were pillars to our research (later mentioned in methodology) as the findings of different action units and emotions are considered culturally universal.

Manual and automated facial coding are reliable, but manual coding is a lot more time consuming (Girard et al., 2013). Learning how to manually code expressions via FACS requires at least 100 h (FACS, n.d.) and analyzing video-recordings takes even longer. To obtain more accurate results needs many more researchers who all have to agree on each part owing to the difficulty of concentrating on long-lasting stimuli and the potential risk of subjectivity—for example the belief that women are more emotional than men (Barrett et al., 1998). On the other hand, automated coding, involving the use of software, may lead to other types of errors. Participants have to be instructed not to shift their attention, cover their faces, or alter their body position during the recording or real-time analysis (Anderson and McOwan, 2006). Manual coders can look out to these things (Kring and Sloan, 1991). In addition, a good quality camera should be used because the lighting conditions are also important (Wesley et al., 2012). A failure to get these right could lead to performance issues with the software. Various studies have found that automated coding has comparable accuracy to manual coding (Girard et al., 2013; Lewinski et al., 2014; Torre et al., 2011), indicating suitability for use in research.

### Vocal cues

Recognizing emotions from speech is not new (Chuang and Wu, 2004; Magdin et al., 2019; Kumbhakarn and Sathe-Pathak, 2015; Rong et al., 2007; Ali et al., 2015). There is substantial evidence showing that emotion affect respiration, articulation, and phonation, which in turn influence the characteristics of the acoustic signal (Banse and Scherer, 1996). The basic acoustic parameters relate to duration, pitch, and intensity, although study findings are contradictory (Rong et al., 2007). In one study higher mean pitch indicates negative emotion (e.g., Stevens and Williams, 1969) but in others it points to positive emotion (e.g., Bezooijen, 1984). Ali et al. (2015) consider pitch essential for classifying emotions—accuracy is significantly higher with pitch (by 20%) than without. Vocal attributes are therefore just as important as facial expressions in expressing emotions (Dasgupta, 2017). Busso et al. (2004) state that voice and facial expressions complement each other, while a combination may considerably improve the accuracy of emotion recognition. The speaker's voice provides information about their age (measured acoustically with automatic age estimation—sound pressure, speech rate, and basic frequency), sex, health, etc. (Schötz, 2007). Relationships between vocal cues, according to Schötz (2007), are complex and influenced by several factors.

### Expressing self-protection

Only one study has been conducted on self-protective vocal cues (Bailey et al., 2022) and one on self-protective facial action units (Bailey et al., 2023). But a number of studies have examined participants' subjective statements during self-protection (Bailey et al., 2020, 2022; Vráblová et al., 2021). Participants emphasized their rights, needs, and their own limits, and gave their experiences meaning but avoided negative feelings and blamed others. Bailey et al. (2022) state that behavioral aspect (in their study) was the most frequent self-protection domain among three constructs analyzed (self-criticism, self-compassion, and self-protection), which leads us to believe that it may be the most expressive construct. Bailey et al. (2023) identified the following facial action units in the self-protective parts of Emotion Focused Therapy sessions: AU1 (Inner Brow Raise), AU4 (Brow Furrow), AU12 (Smirk), and AU18 (Lip Pucker). They discuss the possibility of anger, happiness, contempt, sadness, and fear being linked. They further explain emotions: presumably fear is connected to demanding self-critic (participants felt fear on hearing the demands) while anger and contempt were directed at the inner self-critic in an attempt at assertiveness and a feeling of happiness ensues (having stood up for themselves). Pascual-Leone and Paivio (2013) suggest ways in which protective anger (self-protection) is communicated: outwardly (toward the perpetrator), distinct from other emotions, or in combination with expressing assertiveness. The intensity of the anger should be situation based and with various meanings to be explored. In addition, Pascual-Leone and Greenberg (2005) define self-protection as speaking with loud voice and with moderate/high expressive arousal. In the Bailey et al. (2022) study the self-protective vocal cues of pitch and intensity were higher than those of self-compassion, whilst being very similar to those of self-criticism. Both studies imply that the intensity/amplitude (which influences loudness) and pitch (fundamental frequency which is connected to emotional arousal; Banse and Scherer, 1996) should be higher for more self-protective people during self-protection. Unfortunately, no similar research has been conducted and that particular study was performed in non-laboratory conditions, which limits the application of the findings and there is the possibility of errors.

## The aim of the study

To date there has been insufficient investigation into the vocal cues and facial expressions (emotions, action units) of self-protection, therefore, the investigation is primarily exploratory. By dividing participants into low and high self-protective groups, we can improve self-protection diagnostics. The aim of the current paper therefore was to investigate differences between high and low self-protective participant vocal cues, action units, and emotions. We formulated two hypotheses based on previous studies (mentioned in “Expressing self-protection” section) and one research questions to ensure examination and comparation of high and low self-protective participants in more detail.

H1: High self-protective participants will exhibit anger, contempt, and happiness significantly more often than low self-protective participants (Bailey et al., 2023).

Note that we excluded fear and sadness because these emotions should be linked to preparation stage (hearing demands of self-critic) rather than expressing self-protection as explained by Bailey et al. (2023).

H2: High self-protective participants will have significantly higher pitch and intensity (Bailey et al., 2022; Pascual-Leone and Greenberg, 2007) compared to low self-protective participants.

Q1: How do the facial action units of high self-protective participants differ from those of low self-protective participants?

## Methods

### Measurement instruments

#### Short version of the Scale for Interpersonal Behavior (s-SIB)

The short version of the Scale for Interpersonal Behavior (s-SIB; Arrindell et al., 2002) is based on the 50-item version of the Scale for Interpersonal Behavior (SIB; Arrindell and Ende, 1985). The subscales consist of negative and positive assertion, initiating assertiveness, expression, and dealing with personal limitations. Participants answer the items twice. First they say how nervous or tense they would feel on a scale of 1–5, where 1 means “not at all” and 5 “extremely.” Second, in response to the same item they say how often they behave in the described manner on a scale of 1–5, where 1 means “never” and 5 “always.” Cronbach's alpha for the whole questionnaire is 0.90, for negative statements 0.78, positive statements 0.78, initiating assertiveness 0.76, and expressing and solving personal limitations 0.71. Arrindell and Ende (1985) do not recommend using the total score on its own. They recommend using the subscales and the total score or only score for separate subscales. Higher scores represent more assertive behavior and more adaptive social skills (Parsa et al., 2015). In addition to the short version of the s-SIB scale in Italy (Arrindell et al., 2002), it was validated also in Portugal (Vagos et al., 2014). There is also a Slovak version, which was back translated. Its psychometric properties and factor structure and the norms for the Slovak sample have been reported (Vráblová and Halamová, 2022). Cronbach's alpha (total) for the distress part of the scale was 0.93, of which negative assertion was 0.84, positive assertion 0.83, initiating assertiveness 0.83, and expression and dealing with personal limitations 0.79. For the performance part of the scale, the values are 0.94 (total), negative assertion 0.85, positive assertion 0.82, initiating assertiveness 0.81, and expression and dealing with personal limitations 0.81. Regarding McDonald ω, the bifactor solution was the best fit. Values for both parts (distress and performance) were 0.95, while hierarchical ω was 0.89 for the nervous/tense part and 0.90 for the performance part. In addition, explained common variance (ECV) was over 0.70 in both cases; in the distress part it was 0.73 and in the performance part 0.77.

#### Procedure

The procedure was based on previous research conducted by Whelton and Greenberg (2005) and Kramer and Pascual-Leone (2016). We used the Emotion Focused Therapy (EFT) two-chair technique (Greenberg, 2002). The two chairs help clients to engage with the two sides of the self in dialogue by expressing their thoughts, feelings, needs, etc. (Greenberg et al., 1993). The participants were informed about the possible risks of participation (temporary emotional discomfort) and signed an electronic consent form. After that, they completed the socio-demographic questionnaire and the s-SIB (Arrindell et al., 2002). Participants were then asked to sit on one of the chairs in the room (note that there was only one participant in the room with the researcher). The chairs were positioned opposite each other. The researcher read the imagination of the self-critical moment and participants had 2 min and 30 s in which to recall a similar moment in their recent past (self-critical thoughts about themselves, failure, a specific description of what was happening at that moment). Then the researcher asked the participant to finish the imagination and instructed them to become their inner self-critic for 3 min. After the self-critical monolog, the participant was given the opportunity to respond, again for 3 min and using self-protection. These parts were recorded. Researchers helped participants who found it difficult to continue with the monolog by asking questions such as “*What words do you use to protect yourself/defend your interests when you respond to self-criticism?*” or “*What words do you use to set your boundaries with your self-critic*?” or “*What words do you use to stand up for yourself against your self-critic*?”

### Research sample

To calculate the sample size, we used repeated-measure ANOVA (non-sphericity correction was set to 0.8 and effect size to 0.5—medium effect size). We needed at least 21 participants in each group. Our sample was selected using snowball sampling and based on availability. It consisted of participants scoring in the upper 15th and lower 15th percentiles on the s-SIB Performance Factor, using the Slovak norms for the scale (Vráblová and Halamová, 2022). From a total sample of 239, 33 were high self-protective (11 men, 22 women aged 18–70; M = 32.76; SD = 14.86) and 25 were low self-protective (eight men, 17 women aged 19–49; M = 26.52; SD = 9.25). We did not have missing data as all items were forced to be answered otherwise the participants could not fill out the later questions.

## Data analysis

### Analysis of action units and emotions

The analysis of the action units and emotions was performed using iMotions computer software, version 8.2.4.0 (iMotions). The software is based on the Facial Action Coding System (FACS; Ekman and Friesen, 1978; Ekman, 2002) and identifies the facial expressions or emotions, while pairing the action units with the system (iMotions). iMotions recognizes seven emotions (iMotions):

1. Anger = AU4+AU5+AU7+AU23,
2. Joy = AU6+AU12,
3. Fear = AU1+AU2+AU4+AU5+AU7+AU20+AU26,
4. Contempt = AU12+AU14,
5. Disgust = AU9+AU15+AU16,
6. Surprise = AU1+AU2+AU5+AU26,
7. Sadness = AU1+AU4+AU15.

and 20 action units (iMotions):
AU1 = Inner Brow Raise, AU14 = Dimpler,
AU2 = Brow Raise, AU15 = Lip Corner Depressor,
AU4 = Brow Furrow, AU17 = Chin Raise,
AU5 = Eye Widen, AU18 = Lip Pucker,
AU6 = Cheek Raise, AU20 = Lip Stretch,
AU7 = Lid Tighten, AU24 = Lip Press,
AU9 = Nose Wrinkle, AU25 = Mouth Open,
AU10 = Upper Lip Raise, AU26 = Jaw Drop,
AU12 = Smirk, AU28 = Lip Suck,
AU12 = Smile, AU43 = Eye Closure.

### Analysis of vocal cues

We also conducted a vocal cues analysis of our high and low self-protective participants using the freely available Praat software (Boersma and Weenink). We tried to find something that is relevant to our aims, is easy to use and was also previously used in research. It is compatible with various computer systems, such as the most popular ones—Windows, Macintosh, Linux etc.—and analyzes pitch, intensity, voice breaks, jitter, and shimmer vocal cues. Only the two main commonly used vocal cues were analyzed in this study—pitch and intensity (Boersma, 2013). The recordings were first converted from.mp4 to supported file.wav (Styler, 2013). The audio recordings had to be edited to remove the researcher's voice and other unnecessary content (background noise, silent intervals). For the editing we used the free software Audacity, version 3.2.4 (Team, 2017). Researchers often use Praat in linguistics research (Boersma and Van Heuven, 2001; Styler, 2013) but it can also be used to study emotions (Kumbhakarn and Sathe-Pathak, 2015; Magdin et al., 2019).

### Statistical analysis

The statistical analysis for both the iMotions and Praat was performed in program R—version 4.2.2, package “lme4” (Bates et al., 2015) since our data comprised of repeated calculations of the individuals computed in time. Four different multilevel models were used (2 for iMotions and 2 for Praat). In iMotions, model 1 contained emotion variability (EV), participant variability (PV) and group—high and low self-protective participants (GP). Model 2 contained action unit variability (AV), participant variability (PV) and group—high and low self-protective participants (GP). EV, AV, and PV were set as the random effects and GP as the fixed effect. In model 1, we analyzed the presence or absence of emotion, in model 2 the presence or absence of AU. We therefore used a logistic multilevel regression model, setting the absolute threshold to 50 (more than 50 = 1, < 50 = 0) as recommended in the software manual (iMotions, 2020). Just as iMotions is divided into “emotions” and “action units,” the Praat was divided into two models: “pitch” and “intensity.” Models 3 and 4 contained participant variability (PV) and group—high and low self-protective participants (GP). PV and GR were the random effects. We will provide information about the number of observations, variability of random effect and conditional R2 as well as plotted models.

## Results

### Statistical analysis of emotions

**Figure 1:**
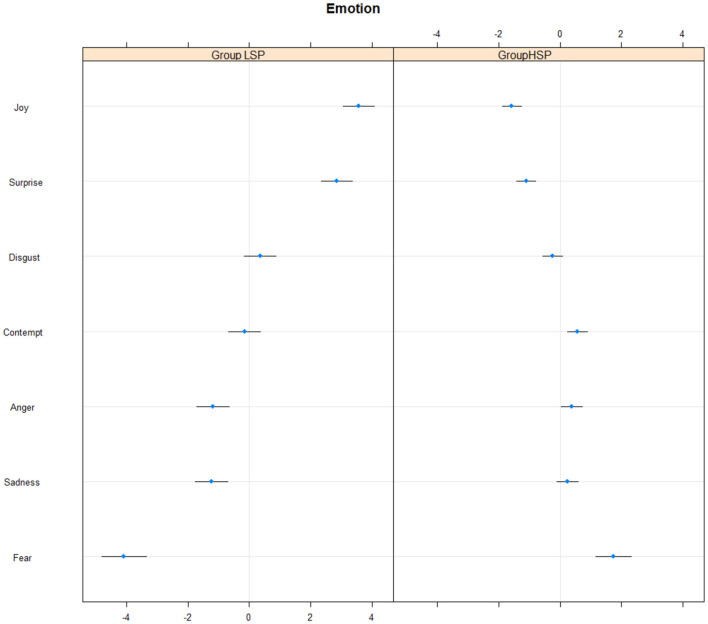
Frequency of emotions in high and low self-protective participants. HSP, high self-protective participants; LSP, low self-protective participants.

There were 1,497,853 observations in the facial analysis of emotions. PV—participant variability was slightly higher (2.58) than EV—emotion variability (5.92). This means that the differences between the emotions were larger than the differences between individuals. Conditional R2 was 0.67, which is considered a high effect size. High self-protective participants exhibited less joy and surprise and more contempt and fear during self-protection compared to low self-protective participants (see Figure 1). Both types of participants displayed lots of joy, surprise, and a little anger, sadness, and fear during self-protective responses to self-criticizing.

### Statistical analysis of action units

**Figure 2:**
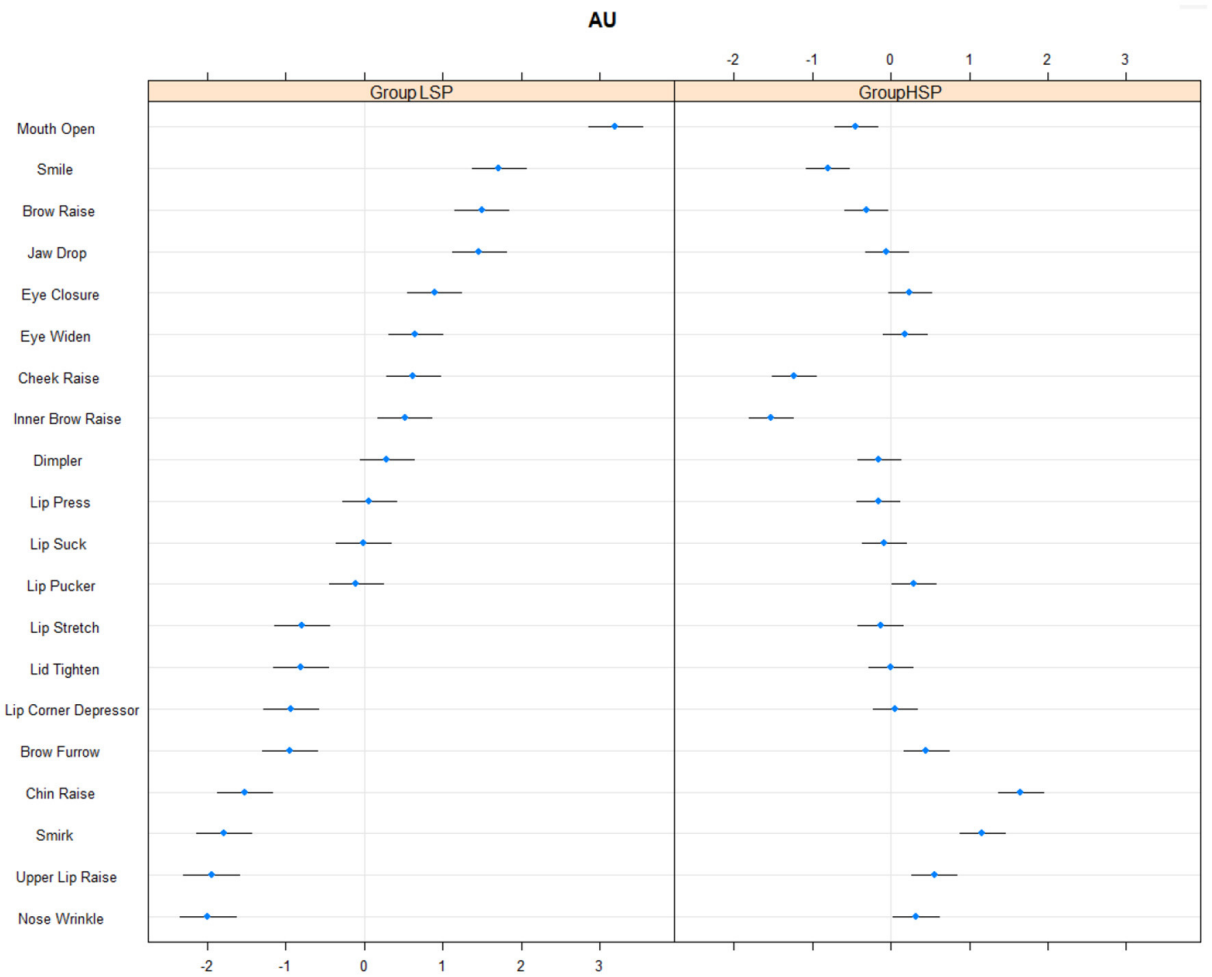
Frequency of action units in high and low self-protective participants. HSP, high self-protective participants; LSP, low self-protective participants.

There were 4,279,580 observations in the facial analysis of action units. PV—participant variability was almost the same (1.68) as AV—action unit variability (1.83). This means that the differences between individuals were slightly lower than the differences between AUs. Conditional R2 was 0.49, which is considered a medium effect size. Compared to low self-protective participants, high self-protective participants used these action units less often: Mouth Open (AU25), Smile (AU12), Brow Raise (AU2), Cheek Raise (AU6), Inner Brow Raise (AU1); and these more often: Brow Furrow (AU4), Chin Raise (AU17), Smirk (AU12), Upper Lip Raise (AU10), and Nose Wrinkle (AU9; see Figure 2). Both types of participants expressed lots of Mouth Open (AU25), Smile (AU12), Brow Raise (AU2), Jaw Drop (AU26), Eye Closure (AU43), Eye Widen (AU5), Cheek Raise (AU6), Inner Brow Raise (AU1), little Lip Stretch (AU20), Lip Tighten (AU7), Lip Corner Depressor (AU15), Brow Furrow (AU4), Chin Raise (AU17), Smirk (AU12), Upper Lip Raise (AU10), and Nose Wrinkle (AU9) during self-protective responses to self-criticizing.

### Statistical analysis of intensity

**Figure 3:**
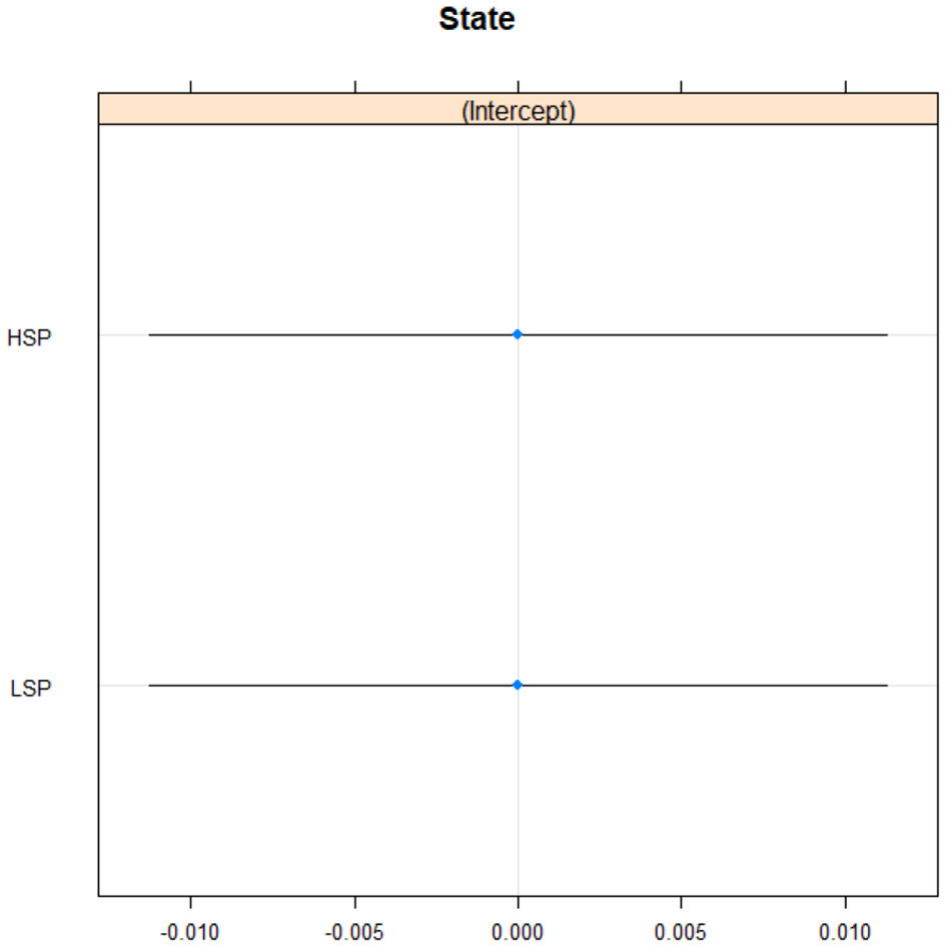
Intensity in high and low self-protective participants. HSP, high self-protective participants; LSP, low self-protective participants.

There were 755,121 observations in the voice analysis of intensity. PT—participant variability was higher (2.807e+01) than GR—group variability (3.300e-05). This means that differences between individual were much greater than the differences between groups. Conditional R2 was 0.10, considered a small effect size suggesting that the differences between groups may not be significant. There were no differences between low and high self-protective participants as seen from Figure 3.

### Statistical analysis of pitch

**Figure 4:**
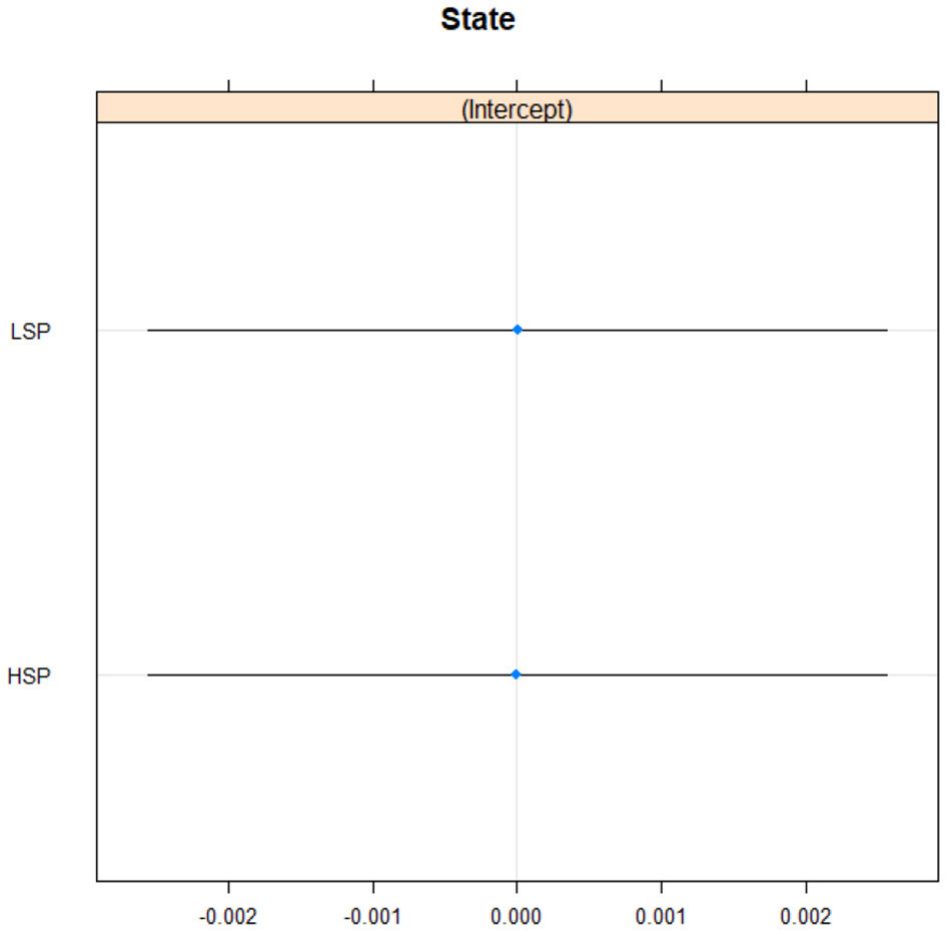
Pitch in low and high self-protective participants. LSP, low self-protective participants; HSP, high self-protective participants.

There were 1,432, 679 observations for the voice analysis of pitch. PT—participant variability was much higher (1.508e+03) than GR—group variability (1.705e-06). This means that the differences between individuals were much greater than the differences between groups. Conditional R2 was 0.45, which is considered a medium effect size. Again, there were no differences between low and high self-protective participants (see Figure 4).

## Discussion

We aimed to ascertain any differences in emotions, action units, and vocal cues between participants with different levels of self-protection (low and high) as current knowledge on this is insufficient. If enough research is conducted to reveal distinct patterns of expressions in high and low self-protective participants, that could improve the diagnosis of individuals with particular levels of self-protection.

In the first hypothesis, we expected that high self-protective participants would be able to stand up for themselves, using the emotions of anger, contempt, and happiness more often compared to low self-protective participants (Bailey et al., 2023). Only contempt was found to be more frequent in high self-protective participants. Surprisingly joy was less frequent. Moreover, fear was also more frequent among high self-protective participants. Therefore, the hypothesis was not supported. We agree with the explanation of Bailey et al. (2023) that participants felt fear when in the preparation stage of taking control and asserting themselves against their demanding self-critic. In our case, contempt, and only contempt, helped our participants to stand up for themselves, and they did not feel joy when fighting their self-critic. They had multiple previous experience of dealing with their inner self-critic and so they were not happy about having to deal with it again. On the other hand, the fact that surprise was less frequent could mean that high self-protective participants are used to confronting their self-critic and so no longer find it surprising. One can assume that low self-protective participants smiled a lot (AU12) because they felt pressurized at finding themselves in an unexpected situation and not knowing how to stand up for themselves. However, by being forced to be self-protective they may have felt happy combating self-criticism and surprised at being able to do so when instructed. The smile action unit is a strong predictor of happiness, while low self-protective participants exhibited cheek raiser (AU6) more frequently as well, providing evidence that the joy was genuine and not faked. Therefore, the low self-protective participants could feel joyful at finding a new effective way to fight their own self-critic, which they had done little before.

There was no support for the second hypothesis that high self-protective participants were more physically expressive (Bailey et al., 2022) and higher in pitch and intensity (Pascual-Leone and Greenberg, 2007). Both high and low self-protective participants were almost identical in expressing vocal cues of pitch and intensity. This could be down to several reasons. The men and women were not separated, but women have a higher pitch than men. The audio recordings varied and there was a big difference in participant age (both these are discussed in the limitations section below). In addition, there was no baseline moment so we do not know if the two groups had higher or lower pitch and intensity than in a neutral situation. It would generally be helpful to know if both groups had high or low arousal, which could improve understanding if future research looks at and compares more groups.

The only research question we asked concerned potential differences in the frequency with which facial action units were exhibited by high vs. low self-protective participants, which had not been previously investigated. We found Brow Furrow (AU4), Chin Raise (AU17), Smirk (AU12), Upper Lip Raise (AU10), and Nose Wrinkle (AU9) to be more frequent in high self-protective participants, and Mouth Open (AU25), Smile (AU12), Brow Raise (AU2), Cheek Raise (AU6), and Inner Brow Raise (AU1) to be less frequent, compared to low self-protective participants. We have already discussed the use of smile and cheek raise in low self-protective participants. In high self-protective participants AU4—Brow Furrow—can mean sadness, fear, and anger according to iMotions (2020). The software more frequently identified fear in high self-protective participants, but there was no difference in anger or sadness between the two groups of participants. This is surprising because AU1 and AU2 were detected less frequently in high self-protective participants, both of which are components of the facial expression fear. The only difference is that these two action units also predict surprise, while AU1 predicts sadness as well. That can be explained by the similarities and differences between fear and surprise. Even Ekman (2003) notes that surprise and fear are very similar and many people have difficulty differentiating between them. Fear is identified by seven action units (1, 2, 4, 5, 7, 20, 26) and surprise by only four (1, 2, 5, 26). AU4 seems to be supported by AU7 (Lid Tighten) and/or AU20 (Lip Stretch) when identified as fear in high self-protective participants. Smirk (AU12) is more frequent in high self-protective participants, which relates to contempt, discussed above, and AU12 supported the results on emotions. Nose Wrinkle (AU9) represents the emotion disgust, but according to the software was not more frequent in either participant group. The supporting action units (15, 16) are ones relating to the lower face, the lips. These action units were not convincing enough to distinguish the groups and so disgust was not more frequent in either of the groups. Some of our participants wore glasses, which can be associated with more frequent use of the “nose wrinkle” action unit, as the participant attempts to adjust the position of their glasses. As there are no basic emotions assigned to Chin Raise (AU17) and Upper Lip Raise (AU10) there is little to say about these results. Finally, Mouth Open action unit (AU25) can be described as the “talking” action unit. High self-protective participants were more efficient at self-protection and had less need to speak to stand up for themselves.

### Limitations

As it has taken almost 4 years to gather enough participants for the analyses, and the COVID-19 pandemic went on for 2 years, this affected the means of data collection. Before and after the pandemic, we collected the data in a lab in the university building, but during the lockdown we had to gather the data online (using either Zoom or MS Teams). Consequently there are differences in the audio and video quality (different microphones, different cameras). Wesley et al. (2012) belief that the accuracy of the facial identification software can be affected by the lighting conditions. We partly resolved this using the editing software (cutting out frozen or disrupted parts, removing background noises, cropping the video etc.). Also, the difference in data collection could cause the participants to behave differently, for example online participants could feel more at ease at home than face-to face participants in an unknown lab with the researcher. This however, was partly managed by the researcher who tried to ease the participants and let them relax before starting the whole process.

Another issue was obtaining a balanced sample. As is often the case in voluntary research (e.g., Nuzzo, 2021; Signorella and Vegega, 1984), there were more women participants in our research, although they did vary in age. Nonetheless neither age or sex could be measured. Another limitation was the possibility of social desirability being expressed. Our participants may have felt that they should behave “appropriately” because they were being recorded. That means they may have altered their expressions according to their beliefs about the social situation at the moment of recording. That can affect the consistency of the results and/or result in the usage of more desirable expressions such as “faked smiles” (Ekman, 2003) and/or voice modulation e.g., pitch tuning.

We highly recommend including a baseline moment in future research. It would be helpful to know what the neutral situation was and to be able to compare it with responses to the self-critic monolog (for both the facial and voice analysis). It could also enable more accurate measurement: facial appearance (Hess et al., 2009) and facial muscle movement (Ekman, 2003) vary from person to person. Additionally, future research could analyze this kind of data in parallel, combining action and vocal cues at the same moment.

## Conclusion

This is the first such study to investigate vocal cues, facial action units, and emotions in high and low self-protective participants. High self-protective participants used more fear and contempt and less joy and surprise compared to low self-protective participants. High self-protective participants used the following more frequently, compared to low self-protective participants, Brow Furrow (AU4), Chin Raise (AU17), Smirk (AU12), Upper Lip Raise (AU10), and Nose Wrinkle (AU9), and used Mouth Open (AU25), Smile (AU12), Brow Raise (AU2), Cheek Raise (AU6), and Inner Brow Raise (AU1) less often. No differences were found in the use of vocal cues. These results give us a better understanding of the construct and could bring us closer to diagnosing people with different levels of self-protection.

## Data Availability

The datasets presented in this article are not readily available because it contains personal data in the form of displaying human faces. Requests to access the datasets should be directed to the corresponding author.
